# Changes in Astroglial K^+^ upon Brief Periods of Energy Deprivation in the Mouse Neocortex

**DOI:** 10.3390/ijms23094836

**Published:** 2022-04-27

**Authors:** Sara Eitelmann, Jonathan Stephan, Katharina Everaerts, Simone Durry, Nils Pape, Niklas J. Gerkau, Christine R. Rose

**Affiliations:** Institute of Neurobiology, Heinrich Heine University Düsseldorf, Universitätsstraße 1, D-40225 Düsseldorf, Germany; sara.eitelmann@hhu.de (S.E.); jonathan.stephan@hhu.de (J.S.); katharina.everaerts@hhu.de (K.E.); simone.durry@hhu.de (S.D.); nils.pape@hhu.de (N.P.); niklas.gerkau@hhu.de (N.J.G.)

**Keywords:** astrocyte, potassium, sodium, pH, extracellular space, ischemia, imaging, ATeam, ion-sensitive microelectrodes, patch-clamp

## Abstract

Malfunction of astrocytic K^+^ regulation contributes to the breakdown of extracellular K^+^ homeostasis during ischemia and spreading depolarization events. Studying astroglial K^+^ changes is, however, hampered by a lack of suitable techniques. Here, we combined results from fluorescence imaging, ion-selective microelectrodes, and patch-clamp recordings in murine neocortical slices with the calculation of astrocytic [K^+^]. Brief chemical ischemia caused a reversible ATP reduction and a transient depolarization of astrocytes. Moreover, astrocytic [Na^+^] increased by 24 mM and extracellular [Na^+^] decreased. Extracellular [K^+^] increased, followed by an undershoot during recovery. Feeding these data into the Goldman–Hodgkin–Katz equation revealed a baseline astroglial [K^+^] of 146 mM, an initial K^+^ loss by 43 mM upon chemical ischemia, and a transient K^+^ overshoot of 16 mM during recovery. It also disclosed a biphasic mismatch in astrocytic Na^+^/K^+^ balance, which was initially ameliorated, but later aggravated by accompanying changes in pH and bicarbonate, respectively. Altogether, our study predicts a loss of K^+^ from astrocytes upon chemical ischemia followed by a net gain. The overshooting K^+^ uptake will promote low extracellular K^+^ during recovery, likely exerting a neuroprotective effect. The resulting late cation/anion imbalance requires additional efflux of cations and/or influx of anions, the latter eventually driving delayed astrocyte swelling.

## 1. Introduction

A classical function of astrocytes is the regulation and maintenance of a low potassium concentration in the extracellular space ([K^+^]_o_) [1]. By taking up K^+^ released from active neurons, astrocytes keep [K^+^]_o_ below the so-called ceiling level of about 10 mM [2,3,4]. This prevents detrimental accumulation of K^+^ in the extracellular space, protecting neurons from excessive K^+^-induced depolarization [5,6]. The mechanisms of astrocytic K^+^ uptake involve plasma membrane transporters as well as K^+^ channels [7,8]. Astrocytic Na^+^/K^+^-ATPase (NKA) apparently plays a predominant role, but the sodium-potassium-chloride cotransporter 1 (NKCC1) is likely to aid K^+^ clearance upon more severe rises in [K^+^]_o_ [9,10].

Maintenance of [K^+^]_o_ by astrocytes is thus largely dependent on their NKA activity and on intact energy metabolism. In the core region of an ischemic stroke, failure of energy metabolism results in a breakdown of astrocyte K^+^ regulation and [K^+^]_o_ homeostasis [5,6,11,12]. A failure of astrocytic K^+^ uptake will not only hamper clearance of extracellular K^+^, but will also cause a loss of K^+^ from astrocytes, contributing to the rise in [K^+^]_o_ and promoting neuronal depolarization during energy failure [13,14]. As opposed to the ischemic core region, available energy resources in the neighboring penumbra may allow full recovery upon timely reperfusion [15]. The penumbra, however, is exposed to repeated waves of spreading depolarizations initiating from the core tissue [16]. Spreading depolarizations impose additional stress onto the cells of the penumbra and promote the gradual expansion of the ischemic core [15,17,18]. They are characterized by transient accumulation of extracellular glutamate, cellular depolarization, loss of cellular ATP, and a reversible increase in intracellular Na^+^ as well as Ca^2+^ concentrations [19,20]. A hallmark and one of the earliest signs of a developing spreading depolarization is an increase in [K^+^]_o_, suggesting that a disturbance in astrocytic K^+^ regulation represents an important initial event [16,21].

Despite its high relevance for the pathogenesis of spreading depolarizations and cell damage upon brain ischemia, quantitative data on astrocytic [K^+^]_i_ during energy failure are extremely rare [12]. This is mostly due to technical hurdles. Available tools include ion-sensitive microelectrodes, which are mostly suited for the measurement of (low) [K^+^]_o_ [22]. A limited toolbox for fluorescence imaging with genetically-encoded or chemical K^+^ indicator dyes exists [23,24,25,26], but these have not yet been successfully employed in astrocytes in situ.

As an alternative to direct measurement, former studies approached astroglial [K^+^]_i_ mathematically, exploiting the dominating plasma membrane permeability of astrocytes for K^+^ (e.g., [14,27,28,29,30,31,32]). In the present study, we followed this approach to provide insights into changes in astroglial [K^+^]_i_ upon brief energy deprivation. We combined different imaging and electrophysiological methods in murine neocortical tissue slices with the calculation of [K^+^]_i_ using a simplified Goldman–Hodgkin–Katz (GHK) equation. Notably, we determined all parameters required experimentally, namely [K^+^]_o_, intracellular and extracellular Na^+^ concentration ([Na^+^]_i_; [Na^+^]_o_), membrane potential (E_M_), and relative Na^+^ over K^+^ permeability (α). This allowed us to estimate astrocytic [K^+^]_i_, as precisely as possible, significantly extending former studies using this strategy.

Our results show an initial rapid loss of K^+^ from astrocytes upon brief energy restriction, confirming earlier reports. In addition, our study demonstrates a biphasic mismatch in the astrocytic Na^+^/K^+^ balance. The initial glial K^+^ loss quantitatively overrides the ischemia-induced increase in [Na^+^]_i_, revealing a substantial negative anion gap during this phase. In contrast, during late recovery from energy depletion, our simulations uncover a transient net gain of astrocytic [K^+^]_i_, reversing the anion gap.

## 2. Materials and Methods

### 2.1. Preparation of Organotypic and Acute Tissue Slices

Acute parasagittal slices containing hippocampus and adjacent somatosensory cortex were obtained from wild type Balb/C mice of both genders at postnatal days (P)6–8 or P14–21. For preparation of organotypic tissue slice cultures, animals at P6–8 were decapitated, their brains immediately dissected and transferred into ice-cold standard artificial cerebrospinal fluid (ACSF) containing (in mM): 130 NaCl, 2.5 KCl, 1.25 NaH_2_PO_4_, 26 NaHCO_3_, 2 CaCl_2_, 1 MgCl_2_, and 10 glucose; pH 7.4, bubbled with carbogen (95% O_2_, 5% CO_2_). The 250 µm-thick slices were then cut using a vibratome (HM650V, Microtome, Thermo Fisher Scientific, Waltham, MA, USA). Slices were transferred to Biopore membranes (Millicell standing insert, Merck Millipore, Burlington, VT, USA) and were kept in an incubator at the interface between humidified air containing 5% CO_2_ and culture medium at 36 °C [33,34] until used in experiments.

For preparation of acute tissue slices of the neocortex, brains of mice at P14–21 were prepared and cut in a modified preparation ACSF, containing 0.5 mM CaCl_2_ and 6 mM MgCl_2_ to dampen glutamate-induced cellular excitation. To label astrocytes, slices were then incubated for 20 min at 34 °C in preparation ACSF containing 0.5–1 µM sulforhodamine (SR) 101, followed by another 10 min at 34 °C in SR101-free standard ACSF [35]. Afterward, slices were kept in standard ACSF at room temperature (20–22 °C) until used for experiments.

Experiments were performed in layers II/III of the somatosensory cortex and were carried out at room temperature. Slices were perfused with ACSF at a rate of 2.5 mL/min. Transient chemical ischemia was induced by perfusing slices with glucose-free standard ACSF containing the cytochrome C oxidase inhibitor sodium azide (NaN_3_; 5 mM) and the non-metabolizable glucose analog 2-deoxyglucose (2-DG; 2 mM) [19,36].

All chemicals were purchased from Merck/Sigma-Aldrich (St. Louis, MO, USA) or AppliChem (Darmstadt, Germany) if not stated otherwise.

### 2.2. Imaging of ATP 

Imaging of intracellular ATP was performed in organotypic slice cultures using the FRET-sensor ATeam1.03^YEMK^ (“ATeam”) [37]. In brief, 0.5 µL of a vector (AAV5/2) carrying the code for ATeam under the control of astrocyte-specific promotor GFAP was applied to the top of a slice after 1–3 days in culture as described before [34]. Slices were maintained in the incubator for a total of at least 10 days before performing experiments.

**Figure 1:**
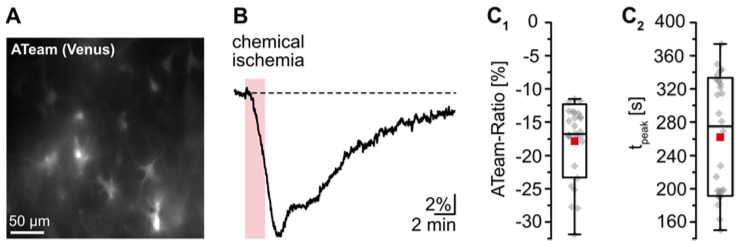
Reduction of astrocytic ATP levels by chemical ischemia for 2 min. (**A**) Image of the Venus fluorescence of ATeam, expressed under the GFAP promotor in an organotypic tissue slice. (**B**) Changes in the ATeam ratio of an individual astrocyte evoked by chemical ischemia (indicated by the red box). (**C**) Box plots of peak changes in astrocytic ATeam ratio (**C_1_**) and the time-to-peak (**C_2_**) upon chemical ischemia. Shown are individual data points (grey diamonds), means (squares), medians (horizontal lines), SDs (boxes), and min/max (whiskers).

Transduced slices were transferred to an epifluorescence microscope (Nikon Eclipse FNI, Nikon, 40× water immersion objective, N.A. 0.8, Tokyo, Japan) equipped with a monochromator (Poly-V; Thermo Fisher Scientific/FEI, Planegg, Germany). ATeam expressed in astrocytes was excited at 435 nm, and images were acquired at 0.5 Hz with a CMOS camera (Orca 4 LT Plus, Hamamatsu Photonics, Herrsching, Germany) (Figure 1A). Emission was split at 500 nm (WVIEW GEMINI optic system; Hamamatsu Photonics, Herrsching, Germany) onto 2 band pass filters (483/32: imaging of eCFP/donor; 542/27: imaging of Venus/acceptor). Fluorescence was collected from regions of interest (ROIs) manually drawn around cell bodies, and the fluorescence ratio (Venus/eCFP) was calculated for individual ROIs. Subsequent analysis was performed offline employing “OriginPro 2021” Software (OriginLab Corporation, Northhampton, MA, USA). Changes in intracellular ATP levels are shown as percentage changes in the Venus/eCFP fluorescence ratio, normalized to the baseline fluorescence ratio before induction of chemical ischemia (“ATeam ratio [%]”).

### 2.3. Imaging of Intracellular Na^+^ and pH

For determination of intracellular ion concentrations in acutely isolated tissue slices, wide field imaging was performed using an upright microscope (Nikon Eclipse FN-1, Nikon, Fluor 40×/0.8 W water immersion objective, Tokyo, Japan) coupled to a Poly-V monochromator (Thermo Scientific/FEI). To measure [Na^+^]_i_, tissue slices were bolus-loaded with the membrane-permeable form of SBFI (SBFI-AM; sodium-binding benzofuran isophthalate-acetoxymethyl ester, 116.7 µM in the ejection pipette; ION Biosciences, San Marcos, TX, USA). SBFI was excited at 400 nm and its fluorescence detected above ~430 nm (409 beam splitter and 510/84 emission filter) [19]. Intracellular pH (pH_i_) was determined upon loading of slices with BCECF (BCECF-AM; 2′,7′-Bis-(2-Carboxyethyl)-5-(and-6)-Carboxyfluorescein-acetoxy-methyl ester, 125 µM; A.G. Scientific, San Diego, CA, USA). BCECF was excited at 458 (isosbestic wavelength) and 488 nm (pH-sensitive wavelength), and its emission was recorded between 511 and 563 nm. Images were acquired at 0.5–1 Hz with an ORCA FLASH 4.0LT camera (Hamamatsu Photonics, Herrsching, Germany).

Fluorescence was collected from ROIs representing cell bodies of SR101-positive astrocytes and analyzed offline employing “OriginPro 2019/2021”. SBFI-emission from individual ROIs was background corrected [38] and corrected for bleaching. For BCECF, the fluorescence ratio (*F*_458_/*F*_488_) was calculated after background correction. Changes in SBFI emission and BCECF ratio were converted into mM [Na^+^] and pH units, respectively, using established in situ calibration procedures [19,38,39].

Baseline [Na^+^]_i_ of layer II/III neocortical astrocytes was determined recently in our laboratory employing an approach introduced by Mondragao et al. [40,41]. This approach was adapted to determine baseline pH_i_. In brief, slices were loaded with BCECF-AM, and the baseline ratio of a selected cell was recorded. Subsequently, this cell was subjected to whole-cell patch-clamp using a pipette solution with a pH of 7.3, which contained 0.5 mM BCECF. The initial baseline pH_i_ of the undisturbed cell was calculated from the change in BCECF ratio upon membrane rupture and the known pH in pipette solution (and calibration parameters determined before).

### 2.4. Measurement of Extracellular K^+^, Na^+^, and pH

[K^+^]_o_, [Na^+^]_o_, and extracellular pH (pH_o_) were measured in acute tissue slices using double-barreled ion-sensitive microelectrodes. These were prepared from two thin-walled borosilicate glass capillaries with filament (GC100F-15, GC150F-15; Harvard Apparatus, Holliston, MA, USA), glued and pulled out together as described before [22]. The tip of one capillary was silanized by exposure to vaporized hexamethyldisilazane (Fluka, Buchs, Switzerland) and filled with a liquid neutral ion carrier based on valinomycin for K^+^ (Ionophore I, Cocktail B, Merck, Darmstadt, Germany), ETH 157 for Na^+^ (Ionophore II, Cocktail A, Merck) or Hydrogen Ionophore I for pH (Cocktail A, 95291, Merck). Afterwards, ion-selective barrels were backfilled with 100 mM KCl (K^+^-sensitive electrodes), 100 mM NaCl (Na^+^), or HEPES (N-(2-hydroxyethyl)piperazine-N′-2-ethanesulfonic acid)-buffered saline (pH), respectively. HEPES-buffered saline contained (in mM): 125 NaCl, 3 KCl, 25 HEPES, 2 MgSO_4_, 2 CaCl_2_, 1.25 NaH_2_PO_4_, and 10 glucose; pH 7.4. Reference electrodes were filled with HEPES-buffered saline.

Calibration of K^+^-sensitive microelectrodes was performed using salines composed of 25 mM HEPES and a total of 150 mM NaCl and KCl, in which [K^+^] was 0–10 mM and [Na^+^] adjusted accordingly. Na^+^-sensitive electrodes were calibrated in salines composed of 25 mM HEPES, 3 mM KCl, and a total of 160 mM NaCl and *N*-methyl-d-glucamine chloride (NMDG-Cl), with [Na^+^] ranging from 70–160 mM and [NMDG^+^] adjusted to maintain osmolarity. Calibration of pH-sensitive microelectrodes was conducted in salines at a pH of 7.0 or 7.6, containing (in mM): 144.25 NaCl (pH 7.0)/108.48 NaCl (pH 7.6), 2.5 KCl, 1.25 NaH_2_PO_4_, and 12 NaHCO_3_ (pH 7.0)/47.77 NaHCO_3_ (pH 7.6), bubbled with carbogen. After calibration, electrodes were positioned in layer II/III at 40–60 µm below the slice surface and [K^+^]_o_, [Na^+^]_o_, or pH_o_ were recorded. Electrodes were calibrated again in the experimental bath directly after each experiment. Data were processed in “OriginPro 2021” and “MS Excel” (Microsoft Corporation, Redmond, WA, USA).

### 2.5. Patch-Clamp Recordings

To measure astrocytic membrane potential (E_M_), patch-clamp recordings were performed at an upright microscope equipped with infrared differential interference contrast (E600FN, Nikon, 60× water immersion objective, N.A. 1.0, Tokyo, Japan) and an infrared video camera (XC-ST70CE, Hamamatsu Photonics, Herrsching, Germany) using an EPC10 amplifier and “PatchMaster” software (Harvard Bioscience/HEKA Elektronik, Lambrecht, Germany). Patch pipettes were pulled from borosilicate glass capillaries (GB150(F)-8P, Science Products, Hofheim am Taunus, Germany) at a vertical puller (PC-10 Puller, Narishige International, London, UK) and had a resistance of 3.5–4.5 MΩ.

For cell-attached recordings [42], pipettes were filled with either ACSF or standard pipette solution. The latter contained (in mM): 116 K-methansulfonate, 32 KCl, 10 HEPES, 8 NaCl, 4 Mg-ATP, and 0.4 Na_2_-GTP; pH 7.3. The offset potential was corrected to account for the potential resulting from concentration differences between pipette solution and ACSF [42]. To record E_M_ in the cell-attached mode, seal resistance must be at least 100-fold higher than the membrane resistance (R_M_) of the recorded cell [42]. R_M_ in cortical astrocytes was around 10 MΩ in 2–3 weeks old animals, and recordings were only accepted if the seal resistance was larger than 1 GΩ, which was continuously monitored using test pulses (−50 pA) every 30 s.

Whole-cell current-clamp recordings were performed to determine the relative Na^+^ versus K^+^ permeability of the plasma membrane (α = P_Na_/P_K_) [43]. To this end, pipettes were filled with standard pipette solution. Voltage traces were sampled at 100 Hz. Measurements were rejected if the seal resistance exceeded 15 MΩ to ensure sufficient electrical and diffusional access to the patched cell. The liquid junction potential was not corrected. Data were analyzed using “IGOR Pro” (WaveMetrics, Lake Oswego, OR, USA), “MS Excel“, and “OriginPro 2021”.

### 2.6. Data Presentation and Statistics

Each set of experiments was performed on tissue slices taken from at least 3 different animals. Results given in the text represent mean ± standard deviation (SD). Data were illustrated in box plots showing individual data points (grey diamonds), mean (square), median (horizontal line), SD (box), and min/max (whiskers). Data were statistically analyzed using “WinSTAT” (R. Fitch Software, Bad Krozingen, Germany) and “OriginPro 2019/2021”. Data were first tested for outliers. Thereafter, normal distribution was assessed using a Kolmogorov–Smirnov test. In case of normal distribution, results were assessed by two-tailed, paired, or unpaired Student’s *t*-tests. Otherwise, results were assessed by the Wilcoxon test and *U*-test (Mann–Whitney) for paired and unpaired data, respectively. *ρ* represents the error probability, * *ρ* < 0.05, ** *ρ* < 0.01, *** *ρ* < 0.001. *n* represents the number of cells or experiments per slice per animal. In case of two comparisons, data were statistically analyzed by the tests described above under post hoc Šidák correction (SC) of critical values [44]: * *p* < 0.0253, ** *p* < 0.005, *** *p* < 0.0005.

## 3. Results

### 3.1. Effect of Transient Energy Depletion on Intracellular ATP

The goal of the present study was to gain insight into changes in astrocytic [K^+^]_i_ upon transient energy depletion, simulating the situation in the ischemic penumbra. Energy failure was induced by transient inhibition of glycolysis and oxidative phosphorylation (“chemical ischemia”) as described earlier [19,36]. To evaluate the effect of brief chemical ischemia on cellular ATP levels, we expressed the nanosensor ATeam1.03^YEMK^ (“ATeam”) in organotypic tissue slices of the neocortex (Figure 1A) [34,37]. Perfusion of slices with the metabolic inhibitors for 2 min caused a well-detectable, reversible decrease in the ATeam ratio by 17.3 ± 5.3% of the baseline level determined in standard ACSF (*n* = 27 cells, 4 tissue slices, 3 animals) (Figure 1B,C; Table 1). Based on our recent calibrations using this sensor [45], these data imply a decline in the intracellular ATP concentration by about 1 mM in response to this manipulation. The ATeam ratio recovered slowly towards baseline, and 15 min after reperfusion with standard saline, ATeam levels were still about 3% lower than the initial baseline value.

These results demonstrate that chemical ischemia induction for 2 min causes a transient decrease in cellular ATP levels in astrocytes. In all following experiments, preparations were perfused with the metabolic inhibitors for 2 min to result in moderate transient metabolic stress.

### 3.2. Changes of Ion Homeostasis and Astrocytic Membrane Potential during Chemical Ischemia

Owing to the lack of suitable techniques for quantitative experimental determination of [K^+^]_i_ in astrocytes in situ, we employed a combined empirical–theoretical approach. The latter exploits the dominating K^+^ permeability of the plasma membrane of astrocytes, which, together with a minor permeability for Na^+^, allows an approximation of their membrane potential (E_M_) by a simplified GHK equation [27,43]:

(1) $$E_{M}=\frac{\mathrm{RT}}{F}\ln\frac{{\left[K^{+}\right]}_{o}+\alpha {\left[{\mathrm{Na}}^{+}\right]}_{o}}{{\left[K^{+}\right]}_{i}+\alpha {\left[{\mathrm{Na}}^{+}\right]}_{i}},$$

where R is the universal gas constant, T is the absolute temperature, F is the Faraday constant, [X^z^]_o/i_ are respective ion concentrations outside and inside of the cell, and *α* is the relative membrane permeability for Na^+^ versus K^+^ (P_Na_/P_K_). Rearranging Equation (1) then enables the calculation of [K^+^]_i_:

(2) $${\left[K^{+}\right]}_{i}=\frac{{\left[K^{+}\right]}_{o}+\alpha {\left[{\mathrm{Na}}^{+}\right]}_{o}}{e^{\frac{E_{M}F}{\mathrm{RT}}}}-\alpha {\left[{\mathrm{Na}}^{+}\right]}_{i}.$$

For a realistic approximation of [K^+^]_i_ using Equation (2), we determined all other parameters ([Na^+^]_i_, [Na^+^]_o_, [K^+^]_o_, E_M_, and α) experimentally in astrocytes in acutely isolated tissue slices, thereby going beyond former studies using this approach (e.g., [14]).

**Figure 2:**
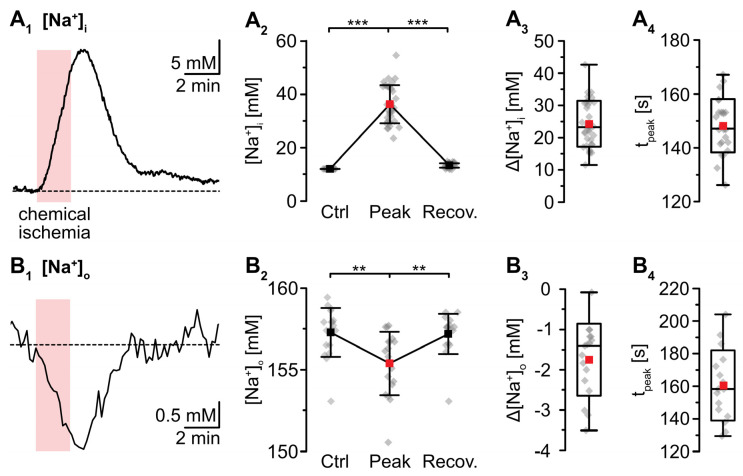
Changes in Na^+^ induced by a chemical ischemia for 2 min. (**A**) Astrocytic [Na^+^]_i_. (**A_1_**): [Na^+^]_i_ increase in an individual astrocyte. (**A_2_**): Plot illustrating baseline [Na^+^]_i_ (data taken from [41]), peak changes in [Na^+^]_i_, and subsequent recovery of [Na^+^]_i_. Shown are individual data points (grey diamonds), means (squares), and SDs (whiskers). (**A_3_**,**A_4_**): Box plots illustrating peak changes in [Na^+^]_i_ (**A_3_**) as well as the time-to-peak (**A_4_**). (**B**) Same illustration as in (**A**) for changes in [Na^+^]_o_. (**B_1_**): Individual measurement. (**B_2_**): Plot illustrating baseline [Na^+^]_o_, peak changes in [Na^+^]_o_, and its subsequent recovery. (**B_3_**,**B_4_**): Box plots of peak changes in [Na^+^]_o_ (**B_3_**) and the time-to-peak (**B_4_**). Box plots in (**A_3_**,**A_4_**) and (**B_3_**,**B_4_**) show individual data points (grey diamonds), means (squares), medians (horizontal lines), SD (boxes), and min/max (whiskers). (**A_2_**,**B_2_**): ** *p* < 0.0005 (Šidák corrected significance level).

Wide field imaging with the Na^+^-sensitive fluorescent dye SBFI was employed to measure [Na^+^]_i_ [38]. Recent work from our laboratory showed that neocortical astrocytes exhibited an average baseline [Na^+^]_i_ of 12.1 mM [41]. Perfusion with metabolic inhibitors for 2 min caused a rapid increase in astrocytic [Na^+^]_i_ by 24.4 ± 7.2 mM (*p* = 2 × 10^−29^, *** after SC) (Figure 2A). Peak [Na^+^]_i_ was reached within 137 ± 21 s and [Na^+^]_i_ declined towards the initial baseline within 10–11 min after reperfusion (*p* = 6 × 10^−28^, *** after SC, *n* = 34/4/4) (Figure 2A_1_,A_2_). In the extracellular space, a baseline [Na^+^]_o_ of 157.4 ± 1.5 mM was determined using ion-sensitive microelectrodes [22]. In response to induction of chemical ischemia, [Na^+^]_o_ decreased by 1.9 ± 1.0 mM (*p* = 0.003, ** after SC) within 158 ± 24 s and then fully recovered within about 3 min upon washout of the drugs (*p* = 0.002, ** after SC; *n* = 22/22/11) (Figure 2B; data summarized in Table 1).

**Figure 3:**
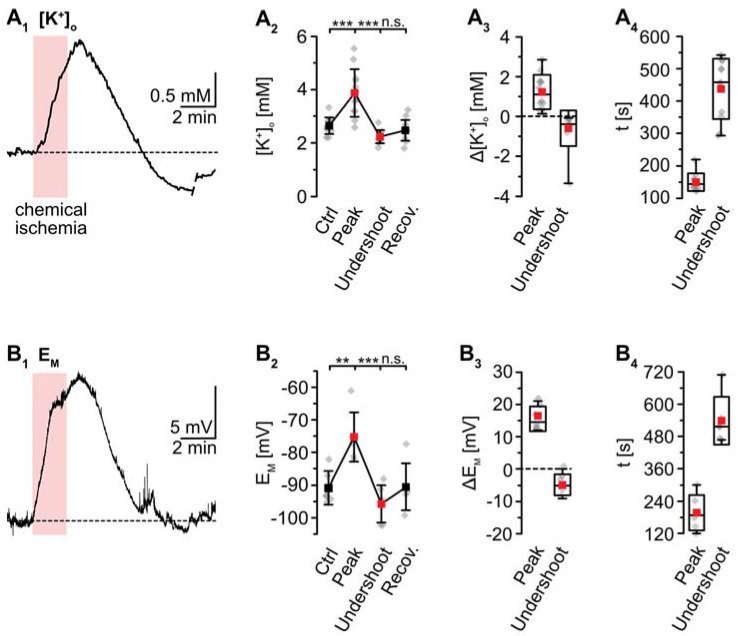
Ischemia-induced changes in [K^+^]_o_ and astrocytic membrane potential. (**A**) [K^+^]_o._ (**A_1_**): Increase in [K^+^]_o_ in an individual measurement. (**A_2_**): Plot illustrating baseline [K^+^]_o_, peak changes in [K^+^]_o_, undershoot and subsequent recovery. Shown are individual data points (grey diamonds), means (squares), and SDs (whiskers). (**A_3_**,**A_4_**): Box plots illustrating peak changes in [K^+^]_o_ (**A_3_**) as well as the time-to-peak and time-to-undershoot (**A_4_**). (**B**) Same illustration as in (**A**) for changes in astrocytic E_M_. (**B_1_**): Individual measurement. (**B_2_**): Plot illustrating baseline E_M_, peak changes in E_M_, hyperpolarization, and subsequent recovery. (**B_3_**,**B_4_**): Box plots of peak changes in E_M_ (**B_3_**) and the time-to-peak and time-to-undershoot (**B_4_**). Box plots in (**A_3_**,**A_4_**) and (**B_3_**,**B_4_**) show individual data points (grey diamonds), means (squares), medians (horizontal lines), SDs (boxes), and min/max (whiskers). (**A_2_**,**B_2_**): ** *p* < 0.005, *** *p* < 0.0005 (Šidák corrected significance levels), n.s.: not significant.

K^+^-sensitive microelectrodes were employed to analyze [K^+^]_o_, revealing a baseline of 2.7 ± 0.3 mM in layer II/III of acute tissue slices (Figure 3). Induction of chemical ischemia for 2 min resulted in a transient increase in [K^+^]_o_ by 1.2 ± 0.9 mM (*p* = 2 × 10^−4^, *** after SC; *n* = 12/12/9) (Figure 3A_1_–A_3_). The maximal [K^+^]_o_ increase was reached within 149 ± 26 s (Figure 3A_4_). This was followed by a long-lasting undershoot of 0.6 ± 0.9 mM, after which [K^+^]_o_ slowly approached the initial baseline (*p* = 5 × 10^−5^, *** after SC) (Figure 3A_2_).

For the determination of α (P_Na_/P_K_), the E_M_ of astrocytes was recorded in the whole-cell patch-clamp mode. In this configuration, astrocytic [Na^+^]_i_ and [K^+^]_i_ were determined by their respective concentrations in the pipette saline (see methods). α was calculated, rewriting Equation (1) as follows and inserting the experimentally determined [Na^+^]_o_ and [K^+^]_o_ (157.4 mM and 2.7 mM, see above):

(3) $$\alpha =\frac{{\left[K^{+}\right]}_{o}+e^{\frac{E_{M}F}{\mathrm{RT}}}*{\left[K^{+}\right]}_{i}}{e^{\frac{E_{M}F}{\mathrm{RT}}}*{\left[{\mathrm{Na}}^{+}\right]}_{i}-{\left[{\mathrm{Na}}^{+}\right]}_{o}},$$

revealing an α of 0.0100 ± 0.0031 in neocortical astrocytes.

As mentioned, whole-cell patch-clamp results in a rapid dialysis of the recorded soma with the pipette saline. This, however, will also result in a partial clamping of intracellular ion concentrations during chemical ischemia, mitigating eventual changes in cellular membrane potentials [14]. To circumvent these effects, measurement of E_M_ induced by metabolic inhibition was performed in the cell-attached mode. After the formation of a GΩ seal, the recorded potential slowly shifted towards more negative values, attaining a stable value of −90.7 ± 5.1 mV within about 10–20 min (*n* = 6/6/4; not shown). Upon perfusion with metabolic inhibitors for 2 min, astrocytes transiently depolarized by 15.5 ± 3.8 mV (*p* = 0.003, ** after SC) (Figure 3B_1_–B_3_). The peak depolarization was reached after 201 ± 65 s (Figure 3B_4_), where after E_M_ showed an undershoot by 4.8 ± 3.2 mV. Subsequently, E_M_ fully recovered to baseline within about 11 min after reperfusion with standard saline (*p* = 4 × 10^−4^, *** after SC) (Figure 3B_1_–B_3_; data summarized in Table 1).

### 3.3. Simulation of Changes in Astrocytic K^+^ Concentration upon Transient Chemical Ischemia

**Figure 4:**
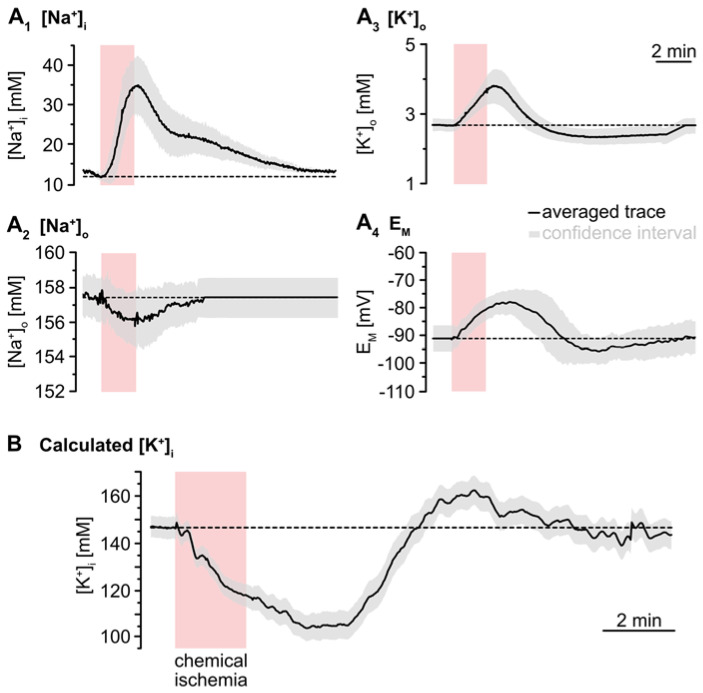
Simulation of ischemia-induced changes in astrocytic [K^+^]_i_. (**A**) Traces show averaged changes of [Na^+^]_i_, [Na^+^]_o_, [K^+^]_o_ and E_M_. The grey envelopes show the 95% confidence intervals. (**B**) The change in astrocytic [K^+^]_i_ upon chemical ischemia was calculated from feeding the average traces shown in (**A**) into Equation (2). The grey envelope again depicts the 95% confidence interval, which was calculated from the individual confidence intervals given in (**A**).

To simulate the time course of astrocytic [K^+^]_i_, the individual traces of the experimentally determined parameters ([Na^+^]_i_, [Na^+^]_o_, [K^+^]_o_, E_M_) were averaged (Figure 4A). The averaged [Na^+^]_o_ trace recovered to baseline within 2–3 min after washout of the inhibitors. As recordings were terminated after this period, the further course of [Na^+^]_o_ was set to this baseline (Figure 4A_2_). In the following, [K^+^]_i_ was calculated for each time point using Equation (2). This revealed an average baseline of astrocytic [K^+^]_i_ of 146.1 mM (Figure 4B). Upon metabolic inhibition, [K^+^]_i_ decreased by 42.8 mM within 229 s to a minimum level of 103.4 mM. After this decline, [K^+^]_i_ slowly increased again, showing a transient overshoot of 16 mM that peaked roughly 6 min after washout. Finally, [K^+^]_i_ slowly recovered to a level close to the initial baseline, which was reached within about 18 min after reperfusion with standard ACSF (Figure 4B; data summarized in Table 1).

### 3.4. Cation–Anion Balance during Transient Chemical Ischemia

Our results presented thus far indicate a mismatch between the calculated maximum loss of K^+^ (43 mM) and the experimentally determined maximum Na^+^ load of astrocytes (24 mM). This apparent cation imbalance requires additional transport of charged molecules across the membrane to maintain electroneutrality. One possibility is the passage of Cl^−^, but recent work indicated that [Cl^−^]_i_ of neocortical astrocytes does not undergo significant changes in response to brief chemical ischemia [36]. An alternative candidate is HCO_3_^−^, for which astrocytes possess efficient transport mechanisms across the plasma membrane [46,47]. The [HCO_3_^−^] is intimately linked to pH via the activity of carbonic anhydrases [48], and we thus studied extra- and intracellular pH to address this possibility.

**Figure 5:**
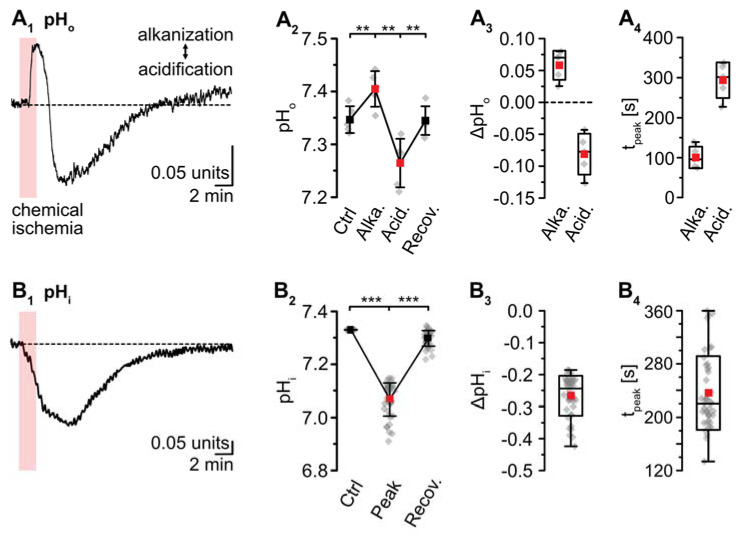

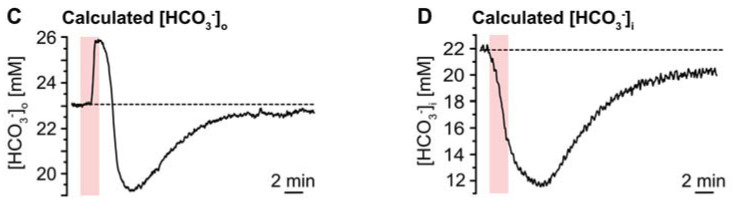
Ischemia-induced changes in pH and [HCO_3_^−^]. (**A**) pH_o_. (**A_1_**): Biphasic change in pH_o_ in an individual experiment. (**A_2_**): Plot illustrating baseline pH_o_, peak alkalinization, acidification, and subsequent recovery of pH_o_. Shown are individual data points (grey diamonds), means (squares), and SDs (whiskers). (**A_3_**,**A_4_**): Box plots illustrating peak changes in pH_o_ (**A_3_**) as well as the time-to-peak (**A_4_**). (**B**) Same illustration as in (**A**) for changes in astrocytic pH_i_. (**B_1_**): Individual measurement. (**B_2_**): Plot illustrating baseline pH_i_, peak changes, and its subsequent recovery. (**B_3_**,**B_4_**): Box plots of peak changes in pH_i_ (**B_3_**) and the time-to-peak (**B_4_**). Box plots in (**A_3_**,**A_4_**) and (**B_3_**,**B_4_**) show individual data points (grey diamonds), means (squares), medians (horizontal lines), SDs (boxes), and min/max (whiskers). (**C**,**D**) Calculated change in [HCO_3_^−^]_o_ and [HCO_3_^−^]_i_, as derived from the Henderson–Hasselbalch equation and Equation (5), respectively. (**A_2_**,**B_2_**): ** *p* < 0.005, *** *p* < 0.0005 (Šidák corrected significance levels).

pH-sensitive microelectrodes revealed a baseline pH_o_ of 7.35 ± 0.03 (*n* = 5/5/4). Chemical ischemia resulted in an alkaline shift of pH_o_ by 0.06 ± 0.02, which peaked at 101 ± 27 s (*p* = 0.016; * after SC; *n* = 5/5/4) (Figure 5A). The initial alkalinization was followed by acidification of pH_o_ to 7.27 ± 0.05 (*p* = 8 × 10^−4^, ** after SC), which peaked at 294 ± 44 s (*n* = 5/5/4) (Figure 5A). pH_o_ slowly recovered to baseline within 13–14 min after washout of the drugs. Astrocytes exhibited a baseline pH_i_ of 7.33 ± 0.26 (*n* = 8/5/4; not shown). pH_i_ dropped by 0.26 ± 0.06 units upon chemical ischemia (*p* = 4 × 10^−43^, *** after SC, *n* = 42/5/5) (Figure 5B). The peak acidification was reached at 237 ± 55 s, after which pH_i_ slowly recovered. At 16–18 min after reperfusion, the pH_i_ was still slightly more acidic than the initial baseline (*p* = 9 × 10^−36^) (Figure 5B_1_,B_2_; data summarized in Table 1).

The Henderson–Hasselbalch equation, which describes the relation between pH and [HCO_3_^−^] was solved for [HCO_3_^−^]_o_ to enable calculation of changes in [HCO_3_^−^]_o_ in response to chemical ischemia as shown in Figure 5C:

(4) $${\left[{{\mathrm{HCO}}_{3}}^{-}\right]}_{o}=10^{{\mathrm{pH}}_{o}-{\mathrm{pK}}_{S\;\left({\mathrm{CO}}_{2}\right)}}*\left[{\mathrm{CO}}_{2}\right].$$

Finally, we used Equation (5) to calculate [HCO_3_^−^]_i_ (Figure 5D) as reported before [49]:

(5) $${\left[{{\mathrm{HCO}}_{3}}^{-}\right]}_{i}=10^{{\mathrm{pH}}_{i}-{\mathrm{pH}}_{o}}*{\left[{{\mathrm{HCO}}_{3}}^{-}\right]}_{o}.$$

**Figure 6:**
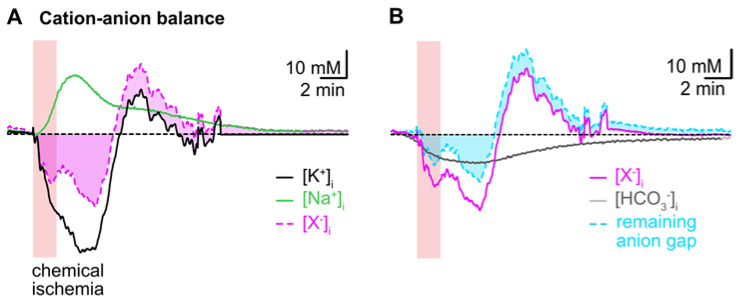
Intracellular cation–anion balance. (**A**) Subtraction of the averaged trace for changes in [Na^+^]_i_ (green trace) from that calculated for [K^+^]_i_ (black trace) reveals a biphasic anion gap (pink trace and area) induced by chemical ischemia. (**B**) Subtraction of the calculated changes in [HCO_3_^−^]_i_ during chemical ischemia (grey trace) reduces the remaining anion gap in the initial phase and increases it in the second phase of recovery (cyan trace and area).

To reveal the presumed contribution of a movement of HCO_3_^−^ to the cation–anion balance during transient chemical ischemia, we first subtracted the averaged traces of astrocytic [Na^+^]_i_ and [K^+^]_i_. This uncovered a biphasic anion gap, predicting an additional loss of anions during the initial decline in [K^+^]_i_, which turned into an anion gain during the overshoot in [K^+^]_i_ (Figure 6A). In a second step, we subtracted the calculated reduction in [HCO_3_^−^]_i_ from the first curve, which reduced the initial anion gap by about 50% as shown in Figure 6B. The ongoing acidification (and ongoing decrease in [HCO_3_^−^]_i_), however, even aggravated the remaining anion gap in the second phase of recovery (Figure 6B).

Taken together, these results indicate that changes in [HCO_3_^−^]_i_ significantly contribute to the changes in the cation–anion balance of astrocytes induced by chemical ischemia. Flux of [HCO_3_^−^]_i_ can, however, not compensate the observed intracellular cation/anion imbalance, suggesting additional movement of other charged molecules across the plasma membrane.

### 3.5. Summary of Results

**Figure 7:**
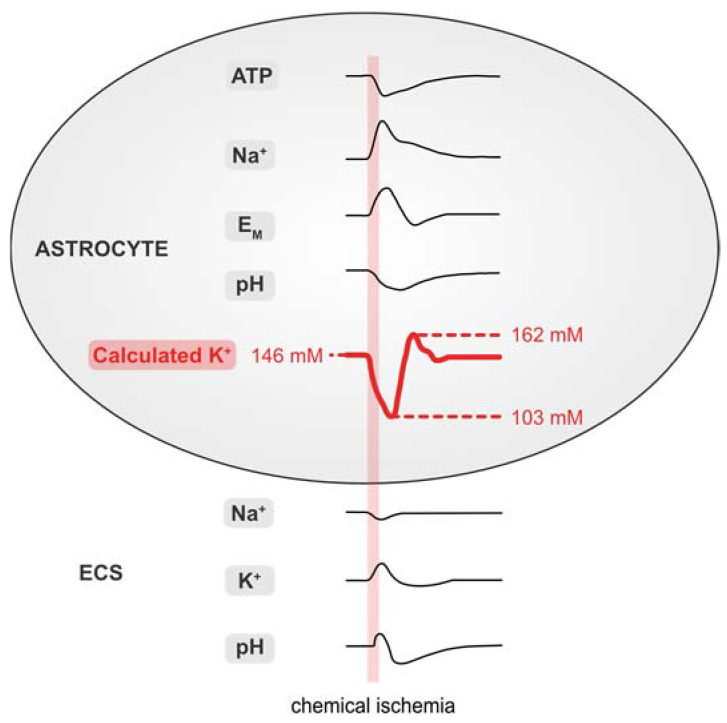
Summary of results. Schematized changes in extra- and intracellular ion concentrations upon chemical ischemia for 2 min. Values for [K^+^]_i_ were approximated from experimental traces using the GHK equation. ECS: extracellular space.

Figure 7 and Table 1 summarize the data presented in this work. Our results demonstrate that brief chemical ischemia, induced by inhibition of cellular metabolism for 2 min, results in a transient decrease in the astrocytic ATP concentration. This is accompanied by a depolarization, an increase in [Na^+^]_i_, and an acidification of astrocytes. At the same time, [Na^+^]_o_ transiently decreases and [K^+^]_o_ increases followed by an undershoot in [K^+^]_o_, while pH_o_ undergoes a biphasic alkaline-acid shift. Calculation of astrocytic [K^+^]_i_ based on these experimentally determined parameters predicts an initial loss of astrocytic [K^+^]_i_, followed by a net, overshooting gain in [K^+^]_i_ during late recovery. Finally, our results show a mismatch of the intracellular cation–anion balance of astrocytes during and after chemical ischemia. This indicates a requirement for additional ion flux across the astrocytic membrane, which is most pronounced in the late recovery phase during which our data predict a net overshoot in [K^+^]_i_.

## 4. Discussion

### 4.1. Baseline [K^+^]_i_ of Astrocytes

While it is widely accepted that glial cells maintain a high [K^+^]_i_ [7,50], direct measurements of astrocytic [K^+^]_i_ are rare due to the lack of suitable techniques. Earlier estimates and measurements in several types of glial cells and based on different indirect methods predicted a baseline [K^+^]_i_ of 100–170 mM [6,27,28,51]. Values determined with ion-selective microelectrodes in rodent glial cells were generally lower [52,53,54,55], which may have been caused by a loss of K^+^ due to impalement with the large-tip electrodes. Employing non-invasive imaging with the fluorescent K^+^ indicator Asante Potassium Green-1, Rimmele et al. determined a [K^+^]_i_ of 133 mM in cultured rat astrocytes [23]. This dye, however, has not been employed for studying [K^+^]_i_ in glial cells in more intact preparations, probably because it does not load well in astrocytes in brain tissue slices (unpublished observations, Rose laboratory). More recently, genetically encoded K^+^ nanosensors were developed, which owing to their low apparent affinities for K^+^, are more suitable for the determination of extracellular [K^+^] [24,25]. A sensor published by Shen et al. enabled the measurement of relative changes in [K^+^]_i_ in HEK cells [26], but its suitability for quantitative imaging of [K^+^]_i_ in brain tissue has not been established yet.

To gain information on [K^+^]_i_ and changes thereof in response to energy deprivation in astrocytes in situ, we, therefore, followed earlier approaches combining experimental determination of different cellular parameters with calculation of [K^+^]_i_ using the GHK equation. This strategy is based on the observation that mature astrocytes express a battery of different K^+^ channels, including inwardly rectifying Kir4.1, the two-pore domain channels (TWIK-1 and TREK-2) as well as large-conductance calcium-activated K^+^ channels (BK_Ca_) [56]. Owing to their high K^+^ permeability at rest [27,57], the membrane potential of astrocytes can be fairly well approximated by a modified GHK equation assuming an additional small Na^+^ permeability [27,43]. Recent work has also demonstrated that short-term ischemic conditions neither alter the intrinsic properties of astrocytic K^+^ channels nor affect the overall membrane conductance, suggesting that their depolarization largely arises from the accompanying changes in [K^+^]_o_ and [K^+^]_i_ [14,58].

The GHK equation evidently also allows for calculation of [K^+^]_i_, provided that all other parameters are known. Here, going beyond earlier reports, we not only measured [K^+^]_o_, [Na^+^]_o_ and E_M_, but also [Na^+^]_i_ as well as the relative Na^+^ permeability in our preparation experimentally, enabling a realistic approximation of [K^+^]_i_. We determined a relative membrane permeability for Na^+^ versus K^+^ (P_Na_/P_K_) of 0.01 in neocortical astrocytes, which is lower than that of astrocytes in the mouse hippocampal CA1 area (0.015; [43]). Under the given conditions and ion concentrations (e.g., a baseline [K^+^]_o_ of 2.7 mM), we obtained a baseline [K^+^]_i_ of 146 mM for neocortical astrocytes. This value is somewhat higher than that reported for astrocytes cultured from the mouse cortex (133 mM) [23]. As in the latter study, astrocytes were bathed in 5.4 mM [K^+^]_o_, they were most likely more depolarized, probably yielding a lower [K^+^]_i_ as compared to our study.

### 4.2. Changes in Astroglial [K^+^]_i_ during Energy Deprivation

A central function of astrocytes is the uptake of K^+^ released by active neurons, thereby controlling and maintaining homeostasis of [K^+^]_o_ in the brain [7,9]. Consequently, several studies reported a rise in astrocytic [K^+^]_i_ in response to neuronal stimulation and/or a rise in [K^+^]_o_ [23,51,52,59]. Glial K^+^ uptake is mainly mediated by the NKA, but other transporters such as the NKCC1 and/or channel-mediated influx may come into play depending on the amplitude and spatio-temporal properties of the [K^+^]_o_ elevation [9,10,60,61,62].

Breakdown of [K^+^]_o_ homeostasis is one of the first consequences of anoxic/ischemic conditions and a restriction in cellular energy supply [13,50,63]; whereas the ischemic core undergoes a complete breakdown of ion homeostasis and massive cell death, recovery is possible in the neighboring ischemic penumbra, which experiences a reduction in cerebral blood flow to 20–40% of normoxic conditions [64]. Repeated waves of spreading depolarizations, however, will impose additional transient ion loads and metabolic stress onto the already stressed cells of the penumbra [15,16,17,18]. Here, we mimicked ischemia-like conditions in the penumbra undergoing waves of spreading depolarizations by brief perfusion of tissue slices with metabolic inhibitors [19,36]. Noteworthy, this chemical ischemia does not represent a complete model of spreading depolarizations developing in the ischemic penumbra. Our experiments, however, confirm a rapid reduction of ATP in neocortical astrocytes after transient inhibition of metabolism, followed by a slow recovery [34]. In a recent study, we performed an in situ calibration of ATeam fluorescence [45]. This was achieved by exposure of organotypic tissue slices to the saponin ß-escin, resulting in a permeabilization of cellular plasma membranes for ATP. Defined changes in the [ATP] then resulted in defined changes in the ATeam FRET-ratio that followed Michaelis–Menten kinetics, revealing an apparent K_D_ of 2.7 mM for ATeam1.03^YEMK^ in both neurons and astrocytes. A linear plot between 1 and 3 mM ATP showed that a 10% change in fluorescence ratio corresponded to a change in [ATP]_i_ by 0.56 mM. Based on this calibration [45], astrocytic ATP exhibited an estimated decrease by about 1 mM in response to chemical ischemia for 2 min in the present study. Such transient, moderate reduction in cellular ATP is one of the hallmarks accompanying the generation of spreading depolarizations [65].

In addition, inhibition of cellular metabolism resulted in a transient decrease in [Na^+^]_o_, an increase in [K^+^]_o_, an increase in astrocytic [Na^+^]_i_, and a depolarization of astrocytes, confirming earlier observations [5,6,13,19,58,66,67]. All changes induced by a chemical ischemia for 2 min recovered towards baseline after washout of the inhibitors, with [K^+^]_o_ and glial E_M_ showing an additional undershoot below baseline and hyperpolarization, respectively [58,67]. As discussed above, ion changes were accompanied by a decrease and subsequent recovery of astrocytic ATP, suggesting a direct correlation between ATP levels and NKA activity, a phenomenon already reported from CA1 pyramidal neurons [68].

Earlier work suggested that the depolarization of astrocytes when exposed to brief periods of ischemic conditions is mainly due to a loss of K^+^ from cells [14,58]. Our simulation based on the GHK equation supports this notion, indicating a decrease in astrocytic [K^+^]_i_ by about 43 mM upon brief inhibition of cellular energy production, most likely as a direct consequence of the decline in cellular ATP and the resulting decrease of NKA activity. Besides channel-mediated loss of K^+^, activation of astrocytic glutamate transporters might, at least partly, contribute to the decline in astrocytic [K^+^]_i_ [19,23].

While initial K^+^ efflux from astrocytes under ischemic conditions will aggravate the accumulation of extracellular K^+^ induced by its release from neurons, our simulation also predicts a delayed gain of astrocytic K^+^ during the late recovery phase. This phenomenon arises from the mismatch in the experimentally determined time courses of the undershoot in [K^+^]_o_ and the astrocytic hyper- and repolarization, respectively. It again suggests ongoing increased activation of the NKA following metabolic inhibition, probably because of the still-elevated [Na^+^]_i_ and/or in response to an increase in [Ca^2+^]_i_ [19,58,60,69]. In addition, astrocytic K^+^ uptake might at least partly be mediated by increased activity of inward NKCC1 [11,36,70].

Notably, the overcompensating uptake of K^+^ by astrocytes reduces [K^+^]_o_ below its initial baseline. The latter will exert a hyperpolarizing effect on neurons and reduce neuronal excitability [8,50], while at the same time increasing the driving force for astroglial glutamate uptake [71]. Astrocytes will thereby exert a neuroprotective role following transient energy depletion through a [K^+^]_o_-mediated dampening of network activity and reduction of neuronal ATP consumption, supporting the recovery of neurons from metabolic stress.

### 4.3. Astroglial Cation–Anion Balance during Energy Deprivation

The K^+^ movements across the astrocytic membrane predicted by our simulation require the additional flux of ions to preserve electroneutrality [6]. While Na^+^ influx compensates for about 50% of the predicted initial loss of K^+^ from astrocytes, it does not fully counteract the simultaneous cation efflux, an observation also reported by earlier studies [6,67]. What is more, owing to its slow recovery, still elevated [Na^+^]_i_ even adds to the delayed predicted gain of K^+^ in the second phase of recovery. This mismatch in the cellular cation–anion balance must be balanced by an additional net gain of cations, followed by additional cation loss in the second recovery phase and/or an initial net loss of anions, followed by a gain of anions. Besides loading with Na^+^, astrocytes are subject to a transient increase in [Ca^2+^]_i_ [19] and [H^+^]_i_ (this study) during brief chemical ischemia. While this might reduce the anion gap in the first phase, the slow recovery from both Ca^2+^ signals and acidification result in its aggravation in the second phase.

A mobile anion which will contribute to the cation–anion balance is HCO_3_^−^ [6]. Our experiments demonstrate a decrease in astrocytic pH_i_ and [HCO_3_^−^]_i_, respectively, in response to chemical ischemia, significantly reducing the predicted imbalance in the first phase. The slow recovery from the intracellular acidification and ongoing decrease in [HCO_3_^−^]_i_, however, will again worsen the anion gap in the second recovery phase. While the initial charge imbalance may thus be compensated for through influx of additional cations (Ca^2+^, H^+^) and a reduction in [HCO_3_^−^]_i_, these processes will later even increase the predicted anion gap.

Cl^−^ is another candidate for compensation of the predicted delayed charge imbalance. Earlier work has demonstrated rapid channel- and transporter-mediated movement of Cl^−^ across astrocytic membranes [72,73,74]. Accumulation of Cl^−^ moreover contributes to astrocyte swelling in ischemic conditions [11,75,76,77,78]. Conversely, a recent study employing fluorescence lifetime imaging in layer II/III astrocytes only reported negligible changes in their [Cl^−^]_i_ in response to a chemical ischemia for 2–5 min [36]. This was probably due to the simultaneous activation of different anion transporters mediating Cl^−^ accumulation (NKCC1) and Cl^−^ efflux (KCC’s and glutamate transporters) [36]. An increase in astrocytic [Cl^−^]_i_ was, however, observed upon a 10 min’ exposure to the metabolic inhibitors, showing that a net gain in Cl^−^ is indeed observed upon more severe conditions (i.e., a larger decline in cellular ATP and/or longer period of energy deprivation). On the other hand, earlier reports demonstrating an apparent mismatch between K^+^ and Cl^−^ movement in astrocytes speculated that in addition to Cl^−^, the net flux of lactate or other anionic osmolytes such as taurine, glutamate or aspartate might help maintaining the cellular cation–anion balance [11,51,79].

While these assumptions are thus supported by earlier work [11,51,79], it should be kept in mind that the proposed anion mismatch was not directly measured but inferred from the calculated [K^+^]_i_. Several factors could have biased our simulation. Among them is the influence of space clamp errors in patch-clamp measurements, a factor especially critical for astrocytes, which not only exhibit a very low input resistance but are also extensively coupled via gap junctions [1,35,56]. Moreover, experimentally determined changes in extracellular ion concentrations might represent underestimates of ion transients occurring in the intact tissue owing to the relatively large tip size of the ion-selective microelectrodes (around 1 µm) [22]. Finally, our intracellular ion measurements represent bulk measurements from cell bodies only, and changes in fine, diffusion-restricted astrocytic processes might be larger in amplitude as well [38,41].

With these considerations in mind, we conclude from our data that the predicted net gain of K^+^ (and positive charge) by astrocytes in the late recovery phase from chemical ischemia will be accompanied by an additional efflux of cations and/or influx of anions. The overshooting uptake of K^+^ by astrocytes will promote and aid the recovery of [K^+^]_o_, thereby exerting a dampening effect on neuronal excitability. Conversely, any net gain of osmolytes by astrocytes will eventually be accompanied by uptake of water, which will promote harmful cell swelling upon prolonged metabolic inhibition [11,75,80]. Identifying the nature of the additional ionic species or charged molecules transported across the plasma membrane is thus highly desirable to shed more light on the mechanisms of astrocytic swelling following brain ischemia [62,76].

## Figures and Tables

**Table 1:** Extra- and intracellular changes induced by chemical ischemia for 2 min.

Parameter	Control	Chemical Ischemia	Δ	*n*	*p*	
[ATP]_o_ [%]	100	82.7 ± 5.3	−17.3 ± 5.3	27/4/3	n.a.	
[Na^+^]_o_ [mM]	157.4 ± 1.5	155.5 ± 1.9	−1.9 ± 1.0 (−1%)	22/22/11	3 × 10^−3^	**
[Na^+^]_i_ [mM]	12.1 ± 2.9 ^§^	36.5 ± 7.2	+24.4 ± 7.2 (+201%)	34/4/4	2 × 10^−29^	***
[K^+^]_o_ [mM]	2.7 ± 0.3	3.9 ± 0.9	+1.7 ± 0.9 (+46%)	12/12/9	2 × 10^−4^	***
[K^+^]_i_ [mM]	146.2	103.4	−42.8 (−29%)	n.a.	n.a.	
E_M_ [mV]	−90.7 ± 5.1	−75.2 ± 7.6	+15.5 ± 3.8 (−17%)	6/6/4	0.003	**
pH_o_	7.35 ± 0.03	7.41 ± 0.03	+0.05 ± 0.03 (+1%)	5/5/4	0.016	*
		7.27 ± 0.05	−0.08 ± 0.03 (−1%)		8 × 10^−4^	**
pH_i_	7.33 ± 0.26	7.07 ± 0.06	−0.26 ± 0.06 (−4%)	42/5/5	4 × 10^−43^	***
[HCO_3_^−^]_o_ [mM]	23.0	25.9	+2.9 (+13%)	n.a.	n.a.	
		19.2	−3.8 (−17%)			
[HCO_3_^−^]_i_ [mM]	22.1	11.6	−10.5 (−52%)	n.a.	n.a.	

Data show peak changes after a 2 min’ chemical ischemia. Note that [K^+^]_o_ moreover undergoes an undershoot and that [K^+^]_i_ overshoots in the recovery phase as described in the results. Data are given as mean ± SD. *n*: number of cells or experiments per slice per animal; *p*: error probability; asterisks depict significance levels after Šidák correction: * *p* < 0.0253, ** *p* < 0.005, *** *p* < 0.0005; n.a.: not applicable; ^§^: from [41].

## Data Availability

The data presented in this study are available on request from the corresponding author.
